# Serum ADAMTS-13 Levels as an Indicator of Portal Vein Thrombosis

**DOI:** 10.1155/2018/3287491

**Published:** 2018-04-18

**Authors:** Tomasz Mikuła, Joanna Kozłowska, Wojciech Stańczak, Mariusz Sapuła, Aleksandra Różyk, Alicja Wiercińska-Drapało

**Affiliations:** ^1^Department of Infectious and Tropical Diseases and Hepatology, Medical University of Warsaw, Warsaw, Poland; ^2^Science Students' Society of the Department of Infectious and Tropical Diseases and Hepatology, Medical University of Warsaw, Warsaw, Poland

## Abstract

**Background:**

Coagulation disorders in patients with liver cirrhosis are a common clinical problem. Cirrhosis should be considered a state of impaired blood clotting or an imbalance of the whole coagulation system. Cirrhosis-induced coagulopathy encompasses disturbances in both the procoagulant and anticoagulant systems. This mechanism may promote the development of thrombosis with portal vein thrombosis (PVT), which is considered an obstacle to orthotopic liver transplantation (OLT). We assessed serum ADAMTS-13 levels in patients with decompensated liver cirrhosis, with and without PVT.

**Material and Methods:**

Serum ADAMTS-13 levels, age, platelet count (PLT), and INR (international normalized ratio) were evaluated in (*n* = 64) patients with liver cirrhosis either with PVT (group 1, *n* = 31) or without PVT (group 2, *n* = 33). The results were compared with those from healthy volunteers (group 3, *n* = 37). Liver cirrhosis was based on Desmet's classification of chronic hepatitis in liver biopsy stage ≥ 3 or liver elastography F-score ≥ 3. Serum ADAMTS-13 levels were measured with Quantikine® ELISA Human ADAMTS13 Immunoassay, R&D Systems Inc. We used Welch's F-test, Games-Howell, one-way ANOVA, Bonferroni test, and logistic regression to determine whether ADAMTS-13 levels were a predictor that was independent of MELD and Child-Pugh scores. All results (*P* < 0.05) were considered statistically significant.

**Results:**

The mean serum ADAMTS-13 level in patients with PVT was significantly lower than that in patients without PVT (*P* = 0.001) and controls (*P* = 0.001). The mean serum ADAMTS-13 level in patients without PVT was significantly lower than that in controls (*P* = 0.001). ADAMTS-13 levels were significantly associated with PVT accounting for the Child-Pugh or MELD score in the logistic regression model.

**Conclusions:**

Low serum ADAMTS-13 levels can be a useful indicator of portal thrombosis in patients with decompensated liver cirrhosis irrespective of Child-Pugh or MELD scores. Further research is needed to determine whether ADAMTS-13 levels will find use in everyday clinical practice.

## 1. Introduction

Liver functions include the synthesis of vitamin K-dependent clotting factors. Thus, an impaired liver function leads to an increased bleeding tendency caused by a deficiency of those factors and of thrombocytes. On the other hand, liver cirrhosis may promote the development of thrombosis, specifically portal vein thrombosis (PVT), which is considered an obstacle in orthotopic liver transplantation (OLT).

Cirrhosis-induced coagulopathy encompasses disturbances in both the procoagulant and anticoagulant systems, rendering compensatory hemostatic mechanisms ineffective in cirrhotic patients. Cirrhosis should be considered a state of impaired blood clotting or, more specifically, an imbalance of the whole coagulation system.

Bleeding esophageal varices and portal hypertensive gastropathy are common complications of advanced, decompensated liver disease. According to population studies, portal vein thrombosis (PVT) is observed in approximately 40% of cirrhotic patients. PVT results in worse clinical outcomes, causes portal hypertension with its complications, and is associated with lower survival of transplant recipients. PVT pathogenesis encompasses local risk factors, such as endothelial activation and stagnant portal blood flow, as well as systemic ones: increased activity of von Willebrand factor, high levels of factor VIII, or reduced antithrombin, protein C, and protein S levels. Anticoagulants, specifically low-molecular-weight heparin, have been proven to be effective in both the treatment and the prevention of PVT [1].

The ADAM (a disintegrin-like and metalloproteinase) proteins, with their subfamily of ADAMTS (a disintegrin-like and metalloproteinase with thrombospondin motif) proteins, are produced exclusively by hepatic stellate cells and play an important regulatory role in coagulation. The ADAMTS proteins are involved in many different processes, such as proteolysis of proteoglycans (ADAMTS-1, 4, 5, 8, and 9) or degradation of cartilage oligomeric matrix protein (ADAMTS-7 and 12).

The ADAMTS-13 protein is an enzyme, Willebrand factor-cleaving protease (vWFCP). In vascular injury, von Willebrand factor (vWF) together with platelets initiates the formation of thrombus. It also serves as a carrier for factor VIII in plasma. Its deficiency is responsible for von Willebrand's disease and can lead to oncogenesis [2, 3].

Functional deficiency of ADAMTS-13 results in the presence of unusually large VWF multimers (UL-VWFMs) in the plasma, which promotes platelet aggregation and thrombus formation. This process is linked to thrombotic thrombocytopenic purpura (TTP), first described by Moschcowitz [4]. TTP is presented as thrombocytopenia and microangiopathic hemolysis, which, if severe, can lead to organ dysfunction. The presence of UL-VWFMs in the plasma is a characteristic feature of this disease [5]. Considering that UL-VWFMs are produced at the sites of vascular injury, ADAMTS-13 deficiency can also be associated with cardiovascular disease, ischemic stroke, and liver disease [6, 7]. In the healthy population, serum ADAMTS-13 levels range from 515 to 1644 ng/mL, SD ± 207 ng/mL (Quantikine ELISA Human ADAMTS13 Immunoassay, R&D Systems Inc.) [8].

## 2. Aim

The aim of this study is to evaluate serum ADAMTS-13 levels as a marker of portal vein thrombosis (PVT) in patients with decompensated liver cirrhosis of various origins.

## 3. Material and Methods

We analyzed (*n* = 64) patients with decompensated liver cirrhosis of various origins (HBV, *n* = 10; HBV/alcohol, *n* = 3; HBV/HCV, *n* = 1; HCV, *n* = 28; HCV/alcohol, *n* = 14; alcohol, *n* = 5; and PBC (primary biliary cirrhosis), *n* = 3) divided into two groups: group 1 with PVT (*n* = 31; 15 females/16 males) and group 2 without PVT (*n* = 33; 14 females/19 males).

The results were compared with those of healthy controls (group 3; *n* = 37; 19 females/16 males). The inclusion criteria for groups 1 and 2 were at least one episode of decompensated liver cirrhosis in the past or present. The exclusion criterion is no history of liver decompensation or other chronic liver diseases. None of the participants of our study had sepsis, DIC, or cancer.

The diagnosis of liver cirrhosis was based on Desmet's classification of chronic hepatitis in liver biopsy specimens (stage ≥ 3) or liver stiffness assessed via elastography (F-score ≥ 3). The presence of portal thrombosis was based on at least two methods of abdominal ultrasonography (USG), computed tomography (CT), or magnetic resonance imaging (MRI).

Serum ADAMTS-13 levels were measured with Quantikine ELISA Human ADAMTS13 Immunoassay, R&D Systems Inc. Furthermore, platelet count (PLT) and INR values were evaluated. We used Welch's *F*-test to determine a possible correlation between ADAMTS-13 levels or INR (i.e., variables that were heteroscedastic according to Levene's test) and PVT status. A post hoc analysis was performed using the Games-Howell test for these two variables: ADAMTS-13 levels and INR. As for age and platelet count (i.e., variables that were homoscedastic according to Levene's test), we used the one-way ANOVA to check for any association between these variables and PVT status. A post hoc analysis was performed using the Bonferroni test. Logistic regression was used to determine whether ADAMTS-13 levels were a predictor that was independent of MELD and Child-Pugh scores. Informed consent was obtained from all individual participants included in the study.

## 4. Results

We found a significant difference in ADAMTS-13 levels between patients with and without PVT (*P* = 0.001). The two groups of cirrhotic patients (with and without PVT) were comparable in terms of age, INR, and platelet count.

The control group in our study exhibited significantly higher mean ADAMTS-13 levels than either of the groups of patients with cirrhosis. As expected, healthy volunteers displayed significantly higher platelet counts and lower INR values than cirrhotic patients, although the control group was younger than the other two (mean age of controls 39.7 years, mean age of patients with PVT 57.7 years, and mean age of patients without PVT 52.6 years; the difference between the control group and either of the other groups was statistically significant, whereas the difference between the two cirrhotic groups was statistically insignificant).

In our study, both Child-Pugh and MELD scores correlated negatively with ADAMTS-13 levels (for ADAMTS-13 and Child-Pugh score: *r* = −0.47 and *P* < 0.01; for ADAMTS-13 and MELD score: *r* = −0.41 and *P* < 0.01). While it is true that Child-Pugh and MELD scores were significantly different in groups 1 and 2, ADAMTS-13 levels were still significantly associated with PVT even in logistic regression models accounting for either Child-Pugh score and ADAMTS-13 levels or MELS score and ADAMTS-13 levels.

All data are presented in Tables 1–5.

## 5. Discussion

It should be noted that serum ADAMTS-13 levels are not routinely measured as part of everyday clinical practice. Despite the various existing studies on this enzyme, which emphasize its role in regulating coagulation or in cardiovascular disease and ischemic stroke pathophysiology, the clinical implications and possible treatment perspectives for this enzyme in hepatology remain not fully understood. Lancellotti et al. assessed serum ADAMTS-13 levels in patients with and without PVT and reported a strong correlation between low ADAMTS-13 levels and the presence of PVT. The study included twenty-four patients with PVT and sixty patients without PVT and demonstrated significantly lower ADAMTS-13 levels in the group with PVT (median 16.8 versus 69.1%, *P* = 0.0047) [9].

These results are consistent with the findings of our study, as we also reported a significant difference in ADAMTS-13 levels between patients with and without PVT (*P* = 0.001). Somewhat different results were reported by Wiese et al., who evaluated serum ADAMTS-13 levels in 61 cirrhotic patients (Child-Pugh classification: class A *n* = 22, class B *n* = 21, and class C *n* = 18) and a control group, consisting of 9 healthy individuals. All participants underwent hepatic vein catheterization to obtain hepatic venous pressure gradient (HVPG); ADAMTS-13 antigen levels were measured in the plasma collected from hepatic veins and a femoral artery. Serum ADAMTS-13 levels were higher in cirrhotic patients than in the control group (*P* < 0.03). However, there was no significant correlation between ADAMTS-13 levels and the Child-Pugh score, HVPG, systemic vascular resistance (SVR), or cardiac output (CO). Furthermore, there was no significant association between serum ADAMTS-13 levels and disease severity or hemodynamic changes [10]. A recent study by Goel et al. analyzed the ADAMTS-13 : vWF ratio in patients with idiopathic noncirrhotic intrahepatic portal hypertension (NCIPH). The authors concluded that ADAMTS-13 deficiency was independently associated with NCIPH [11]. Contrary to an earlier study by Song et al., we reported a correlation between ADAMTS-13 levels and platelet count. This discrepancy in findings may be due to the fact that Song et al. enrolled patients with sepsis- or cancer-induced coagulopathy, whereas our study population is comprised simply of patients with or without PVT [12]. Groeneveld et al. recently published quite a unique study investigating the ADAMTS-13 : vWF ratio in patients after major partial hepatectomy. The findings confirmed that an imbalance of this ratio leads to an increased risk of systemic venous thrombosis or PVT [13].

Similar to our findings, Uemura et al. found a negative correlation between ADAMTS-13 levels and the severity of liver cirrhosis measured in the Child-Pugh score [14].

As the etiology of liver cirrhosis was classified into seven different groups with relatively small group sizes, we did not attempt to determine whether there is an association between ADAMTS-13 levels and the etiology of liver cirrhosis - our study was underpowered to answer these questions.

## 6. Conclusion

The presence of decreased ADAMTS-13 levels in a patient with liver cirrhosis can suggest PVT and warrants further evaluation through medical imaging. However, the findings of this study alone are not sufficient to obviate the need for medical imaging in case of suspicion of PVT, due to the cross-sectional nature of the study and small sample sizes. Further research is needed to determine whether the determination of ADAMTS-13 levels will find use in everyday clinical practice.

## Figures and Tables

**Table 1:** Baseline characteristics of the analyzed groups.

	Patients with PVT Group 1 (*n* = 31) (Mean ± SD)	Patients without PVT Group 2 (*n* = 33) (Mean ± SD)	Healthy controls Group 3 (*n* = 37) (Mean ± SD)
Age (years)	57.7 ± 12.9	52.6 ± 12.6	39.7 ± 8.9
ADAMTS-13 (ng/mL)	695 ± 144	1069 ± 110	1301 ± 203
PLT × 10^3^/*μ*L	116 ± 69	110 ± 63	239 ± 79
INR	1.38 ± 0.29	1.36 ± 0.29	1.04 ± 0.08

**Table 2:** Comparison of the analyzed markers in group 1 (with PVT) and group 2 (without PVT).

	Patients with PVT Group 1 (*n* = 31) (Mean ± SD)	Patients without PVT Group 2 (*n* = 33) (Mean ± SD)	*P* value
Age (years)	57.7 ± 12.9	52.6 ± 12.6	0.23
ADAMTS-13 (ng/mL)	695 ± 144	1069 ± 109	*<0.05*
PLT × 10^3^/*μ*L	116 ± 69	110 ± 63	0.99
INR	1.38 ± 0.29	1.36 ± 0.29	0.96

**Table 3:** Comparison of the analyzed markers in group 1 (with PVT) and group 3 (healthy volunteers).

	Patients with PVT Group 1 (*n* = 31) (Mean ± SD)	Control Group 3 (*n* = 37) (Mean ± SD)	*P* value
Age (years)	57.7 ± 12.9	39.7 ± 8.9	*<0.05*
ADAMTS-13 (ng/mL)	695 ± 144	1301 ± 203	*<0.05*
PLT × 10^3^/*μ*L	116 ± 69	239 ± 79	*<0.05*
INR	1.38 ± 0.29	1.04 ± 0.08	*<0.05*

**Table 4:** Comparison of the analyzed markers in group 2 (without PVT) and group 3 (healthy volunteers).

	Patients without PVT Group 2 (*n* = 33) (Mean ± SD)	Control Group 3 (*n* = 37) (Mean ± SD)	*P* value
Age (years)	52.6 ± 12.6	39.7 ± 8.9	*<0.05*
ADAMTS-13 (ng/mL)	1069 ± 110	1301 ± 203	*<0.05*
PLT × 10^3^/*μ*L	110 ± 63	239 ± 79	*<0.05*
INR	1.36 ± 0.29	1.04 ± 0.08	*<0.05*

**Table 5:** Comparison of Child-Pugh and MELD scores in both groups.

	Patients with PVT Group 1 (*n* = 31) (Mean ± SD)	Patients without PVT Group 2 (*n* = 33) (Mean ± SD)	*P* value
Child-Pugh score	8.0 ± 2.0	6.4 ± 1.3	*<0.05*
MELD score	17.0 ± 7.3	11.9 ± 5.8	*<0.05*
